# Issue Information

**DOI:** 10.1002/psp4.12008

**Published:** 2015-06-24

## Editor-in-Chief

### Piet van der Graaf, PhD, PharmD

Dr. van der Graaf is highly regarded in the areas of pharmacometrics and systems pharmacology as evident through his numerous publications, presentations, and committee memberships. He has a proven track record of innovation in both academic and industrial pharmaceutical research environments and holds several patents in the field of target discovery. He is currently the Director of the Leiden Academic Centre for Drug Research (LACDR) and Professor of Systems Pharmacology at Leiden University (The Netherlands). He received his doctorate training in quantitative pharmacology with Nobel laureate Sir James Black at King’s College London and worked as a postdoctoral fellow with Drs.Meindert Danhof and Douwe Breimer at Leiden University on the development of mechanism-based PKPD approaches. From 1999-2013, he held various scientific leadership roles at Pfizer UK in Discovery Biology, Pharmacokinetics and Drug Metabolism and Clinical Pharmacology/Pharmacometrics. As a member of the Quantitative and Systems Pharmacology Working Group, Dr. van der Graaf co-authored the 2011 NIH White Paper Quantitative and Systems Pharmacology in the Post-genomic Era: New Approaches to Discovering Drugs and Understanding Therapeutic Mechanisms. In 2012 he was elected as Fellow of the British Pharmacological Society.

## Deputy Editor-in-Chief

### Lena Friberg, PhD

Dr. Friberg is an Associate Professor in the Department of Pharmaceutical Biosciences at Uppsala University in Sweden. She holds a Masters in Pharmaceutical Sciences and a PhD from Uppsala University. Her research focuses on pharmacometrics and development of population pharmacokinetic and pharmacodynamic models for desired effects and adverse events in a range of therapeutic areas, such as oncology, bacterial infections, schizophrenia, rheumatoid arthritis, and QT prolongation. Currently, most of her research is focused on development of PKPD-models for quantitative, longitudinal predictions of use for translation of drug effects from preclinical (in vitro and in vivo) to patients and fromearly to late clinical development.

## Associate Editors

### Stephen Duffull, PhD

Stephen Duffull is a Professor of Clinical Pharmacy and Dean of the School of Pharmacy at the University of Otago, Dunedin, New Zealand. He runs a modeling and simulation lab within the School of Pharmacy. Research interests include optimal design, MCMC methods particularly in clinical toxicology and haemostasis. He has been involved in the area of PKPD and nonlinear mixed effects modeling for 20 years. Currently his research involves the development of pharmacological models, modeling of data and the design of clinical studies. These foci essentially revolve around the therapeutic areas of coagulation, malaria and clinical toxicology.

### Douglas A. Lauffenburger, PhD

Dr. Lauffenburger is Ford Professor of Bioengineering and Head of the Department of Biological Engineering at MIT, and also holds appointments in the Department of Biology and the Department of Chemical Engineering. He is a member of the Koch Institute for Integrative Cancer Research, the Center for Biomedical Engineering, the Center for Environmental Health Sciences, and the Center for Gynepathology Research, and is Director of the Computational & Systems Biology Initiative. He earned BS and PhD degrees in chemical engineering from the University of Illinois and the University of Minnesota, in 1975 and 1979, respectively. His major research interests are in cell engineering: the fusion of engineering with molecular cell biology. Dr. Lauffenburger is a member of the National Academy of Engineering and of the American Academy of Arts & Sciences, and has served as President of the Biomedical Engineering Society, Chair of the College of Fellows of AIMBE, and on the Advisory Council for the National Institute for General Medical Sciences at NIH.

### Lang Li, PhD

Lang Li, PhD, is an Associate Professor in the Department of Medical and Molecular Genetics in the Indiana University School of Medicine (IUSM). He received his PhD in Biostatistics from the University of Michigan in Ann Arbor, Michigan, before joining the IUSM in 2001. Dr. Li is the Interim Director of the Center for Computational Biology and Bioinformatics in the IUSM, and the Associate Director of the Indiana Institute of Personalized Medicine (IIPM). He uses informatics, genomics and statistics to investigate drug efficacy and safety problems. He is interested in both the molecular mechanisms and clinical significance of drug safety and efficacy.

### DonaldMager, PharmD, PhD

Dr. Mager is an Associate Professor of Pharmaceutical Sciences at the University at Buffalo, State University of New York (UB). He received his BS degree in Pharmacy from UB in 1991, followed by the PharmD (2000) and PhD (2002) degrees. Prior to joining the faculty at UB, he completed two years of postdoctoral training as an IRTA Fellow at the National Institute on Aging of the NIH, where he continued to serve as a special volunteer from 2004 to 2010. He received the University at Buffalo Young Investigator Award in 2006 and the New Investigator Award in Pharmacokinetics, Pharmacodynamics, and Drug Metabolism from AAPS in 2007. He currently serves on the Clinical Pharmacology Advisory Committee to the FDA and the Editorial Advisory Boards of Biopharmaceutics and Drug Disposition and Journal of Pharmacokinetics and Pharmacodynamics. He was also elected as a Fellow to the American College of Clinical Pharmacology, as Chair of the Clinical Pharmacology and Translational Research section of AAPS, and as a member of the Board of Directors to the International Society of Pharmacometrics. His research focuses on identifying molecular and physiological factors that control the pharmacological properties of various drugs including anti-cancer, immunomodulatory, and anti-platelet drugs.

### France Mentré, MD, PhD

Dr. Mentré is a Professor of Biostatistics in the School of Medicine of University Paris Diderot (Paris 7). She heads an INSERM research team on Biostatistical Modelling and Pharmacometrics. She has worked on development and application of methods for nonlinear mixed-effects models in pharmacokinetics and pharmacodynamics for more than 25 years. She is involved in the development of the software PFIM for optimal design. Dr. Mentré has co-authored more than 150 articles in biostatistics, pharmacometrics, clinical pharmacology and medicine.

### Amin Rostami-Hodjegan, PharmD, PhD

Dr. Rostami-Hodjegan is a Professor of Systems Pharmacology at the Centre for Applied Pharmacokinetic Research (CAPKR) in the School of Pharmacy and Pharmaceutical Sciences at the University of Manchester. He has an active program of training PhD students in CAPKR in Systems Pharmacology and Pharmacokinetics, and numerous students graduated under his supervision from the University of Sheffield, where he was a Professor of Systems Pharmacology before joining the University of Manchester in 2009. As the Vice President of Research & Development at Simcyp Limited (a Certara Company), Dr. Rostami-Hodjegan leads a team of over 30 scientists working on extrapolation of in vitro data on drug metabolism to predict in vivo pharmacokinetics and pharmacodynamics in virtual patient populations. Professor Rostami-Hodjegan has authored/co-authored over 120 peer-reviewed full articles and serves on the Editorial Boards of several journals. He has been an invited speaker at over 100 national and international meetings and has led a number of hands-on workshops in the area of in vitro-in vivo extrapolation as applied to ADME in Drug Development.

### Paolo Vicini, PhD

Dr. Vicini is a Research Fellow in the Pharmacokinetics, Dynamics and Metabolism Department at Pfizer Worldwide Research and Development in San Diego, CA. He received his PhD in bioengineering from the Polytechnic of Milan in 1996. In 2003, he received the IEEE Engineering in Medicine and Biology Early Career Achievement Award. His previous research focus was on the development of data analysis software and the application of modeling and simulation to glucose and insulin metabolism, cancer, pharmacokinetics and pharmacodynamics. Since his move to Pfizer in 2008, his work has focused on translational modeling and simulation in support of various discovery programs. He has published more than 100 articles employing diverse modeling and simulation technology on a variety of applications.

## Editorial Board

**Sandra Allerheiligen, PhD**

Merck Research Laboratories

West Point, PA, USA

**Ioannis Androulakis, PhD**

Rutgers University Surgery, UMDNJ-RWJ Medical School

Piscataway Township, NJ, USA

**Joseph P Balthasar, PhD**

University of Buffalo

Buffalo, NY, USA

**JeffreyS. Barrett, PhD, FCP**

The Children’s Hospital of Philadelphia

Philadelphia, PA, USA

**Robert Bies, PharmD, PhD**

Indiana University

Bloomington, IN, USA

**Kim L. R. Brouwer, PharmD, PhD**

University of North Carolina at Chapel Hill

Chapel Hill, NC, USA

**Jenny Chien, PhD**

Eli Lilly & Co.

Clinton, IN, USA

**DavidZ. D’Argenio, PhD**

University of Southern California

Los Angeles, CA, USA

**Oleg Demin, PhD**

CSO Institute for Systems Biology SPb

Moscow, Russia

**Hartmut Derendorf, PhD**

University of Florida

Gainesville, FL, USA

**JamesM. Gallo, PharmD,PhD**

Mount Sinai School of Medicine

New York, NY, USA

**Christine Garnett, PhD**

Certara

St Louis,MO, USA

**David Gavaghan, PhD**

University of Oxford

Oxford, United Kingdom

**Ekaterina Gibiansky, PhD**

QuantPharm LLC

North Potomac,MD, USA

**Bart Hendriks, PhD**

Merrimack Pharmaceuticals

Cambridge,MA, USA

**Nicholas H.G. Holford, MBChB**

The University of Auckland

Auckland, New Zealand

**Matt Hutmacher, PhD**

Ann Arbor Pharmacometrics Group

Ann Arbor,MI, USA

**Ravi Iyengar, PhD**

Mount Sinai School of Medicine

New York, NY

**Jin Yan Jin, PhD**

Genentech, Inc

San Francisco, CA, USA

**Mats Olof Karlsson, PhD**

Uppsala University

Uppsala, Sweden

**Terrence Kenakin, PhD**

University of North Carolina, Chapel Hill

Chapel Hill, NC, USA

**Hiroaki Kitano, PhD**

The Systems Biology Institute

Tokyo, Japan

**Charlotte Kloft, PhD**

Freie Universitaet Berlin

Berlin, Germany

**Joerg Lippert, PhD**

Bayer Healthcare Pharmaceuticals

Leverkusen, Germany

**Phil Lowe, PhD**

Novartis Pharma AG

Basel, Switzerland

**Wei Lu, PhD**

Peking University/Pfizer Pharmacometrics

Beijing, China

**EfthymiosManolis,MSc**

EuropeanMedicines Agency

London, United Kingdom

**PeterMilligan, PhD**

Pfizer Global Research and Development

London, United Kindgdom

**Diane R Mould, PhD, FCP**

Projections Research Inc.

Phoenixville, PA, USA

**Jerry Nedelman, PhD**

Novartis Pharmaceuticals Corporation

East Hanover, NJ, USA

**Kyungsoo Park,MD, PhD**

Yonsei University College of Medicine

Seoul, Republic of Korea

**Marc Pfister, MD, FCP**

University of Basel

Basel, Switzerland

**Jose Pinheiro, PhD**

Johnson and Johnson Pharmaceutical Research

Raritan, NJ, USA

**MalcolmRowland, DSc, PhD**

University of Manchester

Manchester, United Kingdom

University of California, San Francisco

San Francisco, CA, USA

**Radojka Savic, PhD**

University of California

Oakland CA, USA

**Virginia D. Schmith, PhD, FCP**

GlaxoSmithKline

Zebulon, NC, USA

**Vikram Sinha, PhD**

US Food and Drug Administration

Silver Spring,MD, USA

**Brian Smith, PhD**

Amgen

Thousand Oaks, CA, USA

**Peter Sorger, PhD**

HarvardMedical School

Cambridge,MA, USA

**Yuichi Sugiyama, PhD**

Riken Innovation Center

Sugiyama Laboratory

Yokohama, Japan

**Joel Tarning, PhD**

Mahidol-Oxford TropicalMedicine Research Unit

Bangkok, Thailand

**Nicholas Tatonetti, PhD**

Columbia University

New York, NY

**An Vermeulen, PhD**

Janssen Research and Development

Beerse, Belgium

**Alexander A. Vinks, PharmD, PhD, FCP**

University of Cincinnati, College ofMedicine

Cincinnati, OH, USA

## Overview

*CPT: Pharmacometrics & Systems Pharmacology* (CPT:PSP) is an official open access journal of the American Society for Clinical Pharmacology and Therapeutics (ASCPT).

*CPT: Pharmacometrics & Systems Pharmacology* is a cross-disciplinary journal devoted to publishing advances in quantitative (e.g., modeling and simulation) methods as applied in pharmacology, physiology and therapeutics in humans. The journal welcomes original research articles, reviews and tutorials that bridge the following areas: pharmacometrics, modeling and simulation as applied to the design and evaluation of clinical trials, systems pharmacology modeling, particularly with a mechanistic link to human (patho)physiology, disease modeling, “population” or mixed-effects pharmacokinetics and pharmacodynamics (PKPD) modeling, modeling and simulation to support translational research, physiologically-based pharmacokinetics (PBPK), modelbased meta-analyses of clinical trials, mechanism-based pharmacokinetic-pharmacodynamic modeling, computational pharmacology, bioinformatics, comparative efficacy, effectiveness and cost-effectiveness. Systems pharmacology may involve the application of systems biology approaches to study drug activities, targets and effects. The discipline is often defined with reference to engineering and pharmacological principles as the quantitative analysis of the dynamic interactions between drugs and a biologic system that aims to understand the behavior of the system as a whole. The common focus will be on quantitativemethods that improve our understanding of pharmacology and therapeutics in humans.

## Abstracting and Indexing

- Embase (Elsevier)
- SCOPUS (Elsevier)

## Society Information

Founded in 1900, the American Society for Clinical Pharmacology and Therapeutics (ASCPT) is a scientific society dedicated to improving the understanding and use of existing drug therapies and developing better and safer treatments for the future. With more than 2,000 professionals whose primary interest is to promote and advance the science of human pharmacology and therapeutics, the society is the largest scientific and professional organization serving the discipline of clinical pharmacology. To learnmore, or to apply for membership, visit: www.ascpt.org

**All articles published in this journal are made available under the terms of the Creative Commons Attribution License. Permission is therefore not required for academic or commercial reuse, provided that full attribution is included in the new work.**

## Disclaimer

The Publisher, Society and Editors cannot be held responsible for errors or any consequences arising from the use of information contained in this journal; the views and opinions expressed do not necessarily reflect those of the Publisher, Society and Editors, neither does the publication of advertisements constitute any endorsement by the Publisher, Society and Editors of the products advertised.

## Publisher

*CPT: Pharmacometrics & Systems Pharmacology* is published by Wiley Periodicals, Inc., 350 Main Street, Malden, MA 02148; Telephone: 781-388-8200; Fax: 781-388-8210.

